# Plasticity of nanocrystalline alloys with chemical order: on the strength and ductility of nanocrystalline Ni–Fe

**DOI:** 10.3762/bjnano.4.63

**Published:** 2013-09-19

**Authors:** Jonathan Schäfer, Karsten Albe

**Affiliations:** 1 Technische Universität Darmstadt, Fachbereich Material- und Geowissenschaften, Fachgebiet Materialmodellierung, Petersenstr. 32, D-64287 Darmstadt, Germany

**Keywords:** nanocrystalline materials, grain boundary structure, grain boundary segregation, plastic deformation, molecular dynamics

## Abstract

Plastic deformation and alloying of nanocrystalline Ni–Fe is studied by means of atomic scale computer simulations. By using a combination of Monte-Carlo and molecular dynamics methods we find that solutes have an ordering tendency even if grain sizes are in the nanometer regime, where the phase field of the ordered state is widened as compared to larger grain sizes. Tensile testing of disordered structures with various elemental distributions and the simultaneous analysis of intragranular defects reveal that solid solution strengthening is absent for the studied grain sizes. The composition and relaxation state of the grain boundary control the strength of the material, which is also found for ordered structures (L1_2_), where dislocation activity is suppressed.

## Introduction

In intermetallics grain refinement to the nanometer scale has been considered as a possible route for achieving room temperature ductility in this otherwise brittle class of materials [1–2]. The underlying assumption is that for very small grain sizes plasticity can be carried by grain boundary (GB) mediated processes rather than by energetically expensive superlattice dislocations [3–4].

The experimental realization of a nanocrystalline (nc) microstructure of an ordered alloy, however, strongly depends on the route of preparation. For electrodeposited nc Ni–Fe alloys (up to 28% Fe) a solid solution with no chemical order was observed [5]. In Ni_3_Al, a partially ordered state was found after rolling at liquid nitrogen temperature to obtain a nanometer grain size [6]. In nanostructured Ni_3_Al processed by ball milling [7] or high pressure torsion [8], on the contrary, a complete loss of order is observed during preparation. Grain refinement by severe plastic deformation (SPD) of B2 FeAl leads to a partial destruction of the long range order and the formation of ordered nanodomains, which can grow to the size of grains upon heating and restore the order [9].

Concerning the mechanical properties of nc intermetallics, grain refinement often leads to a severe strengthening but does not increase the room temperature ductility [10]. It was demonstrated experimentally, that additional modifications, e.g., a bimodal grain structure and nanotwins can enhance the ductility while conserving the strength [10]. For nc Ni–Fe alloys, several observations were made: For nc disordered Ni–Fe, prepared by electrodeposition, where the Fe content was modulated to control the grain size, an inverse Hall–Petch relationship was reported with a critical grain size around 15 nm [5]. Nc disordered Ni–Fe (5.6% Fe) with an average grain size of 10 nm showed a tensile yield strength in the order of 2 GPa and an increase in strength after annealing even though some grain growth was observed [11]. It was reported that plastic deformation processes in nc Ni–Fe (15% Fe) undergo a transition with applied strain, where at low strains the strain is mainly accommodated by the grain boundaries while at large strains dislocation motion becomes dominant [12].

Computational attempts such as molecular dynamics simulations (MD) can help to understand the macroscopic properties by means of the atomistic processes. For intermetallic phases, MD simulations have been successfully employed, to investigate, e.g., the nucleation of dislocations from surfaces [13] or at GBs [14]. Furthermore, the mechanical response of intermetallic nanostructures such as nanowires has been studied by MD [15].

For understanding the atomistic processes that are carrying and controlling plastic deformation in nc intermetallics (Ni–Fe), we present a set of MD simulations. Samples with 5 and 15 nm average grain size are constructed according to the Voronoi tessellation method [16], then chemically equilibrated via a hybrid MD/Monte-Carlo (MD/MC) scheme and finally tested in tensile straining tests. By this scheme we are able to generate samples of different compositions and a varying internal order for a given average grain size. This allows to explore their respective influence on the macroscopic strength. We perform a detailed analysis of the virtual structures and investigate the influence of composition, element distribution and the degree of order on the interplay of intergranular and intragranular deformation mechanisms.

## Methodology

We employ a hybrid simulation method [17] that has been used for describing miscible random alloys [18] as well as segregating systems [19]. The atomic interaction is modeled by an embedded-atom (EAM) type potential which reproduces the phase diagram of Ni–Fe [20]. The hybrid method allows for structural relaxation by MD and for chemical equilibration by interleaving MC steps, yielding structurally equilibrated samples with an equilibrium distribution of the constituents. The MC algorithm, which is sampling a semi-grandcanonical ensemble [17–18,21], equilibrates the distribution of the constituents and the composition of the system for a given difference of chemical potentials Δμ = μ_Ni_ − μ_Fe_. The method is used within the framework of the freely-available molecular dynamics code LAMMPS [22], which was extended to perform the MC steps [17].

Initially, we created nc model structures of perfectly (L1_2_) ordered FeNi_3_ with average grain sizes of 5 and 15 nm consisting of 432 and 54 grains, respectively. The Voronoi tesselation method [16] was used to set up the grain shapes based on randomly placed center points in 3-dimensional periodic cubic simulation box. The lattice orientations of the grains were taken from a random isotropic distribution. For avoiding spurious configurations in the as-prepared Voronoi samples, we deleted atoms from the grain boundaries that were closer than 2.0 Å to other atoms prior to relaxation. Relaxation and equilibration of the composition and distribution of the constituents was then performed at 600 K for 1 ns at zero hydrostatic pressure using Berendsen’s [23] thermostat and barostat. During this MD equilibration, one full MC step was performed every 40 fs, i.e., 25,000 trial exchanges were performed on each atom in the system on average. For obtaining the equilibrium chemical distribution at a given composition, the temperature parameter for the MC algorithm was the same as in the MD stage. For the variation of the global composition, we choose different values for Δμ to chemically equilibrate the system at a global composition, deviating from the stoichiometric concentration.

The short range order in the system was evaluated by computing the Warren and Cowley [24] order parameter for each atom surrounded by its 12 nearest neighbors as α*_i_* = 1 − *Z_j_*/(12 × (1 − *c_i_*)), where α*_i_* denotes the ordering parameter for atom of type *i*, *Z_j_* is the number of atoms of the according other type among the 12 nearest neighbors and *c_i_* is the global concentration of atoms of type *i*. For distinguishing atoms located in grain boundaries from those in the bulk, we use the common neighbor analysis (CNA) [25]. The cutoff parameter *R*_CNA_ for identifying nearest neighbors was chosen between the first and second nearest neighbor shells: 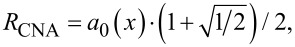 with *a*_0_(*x*) being the static lattice parameter for a given concentration *x*. Analysis of the defects within the microstructure was done by a novel algorithm, which allows for the extraction and analysis of dislocations from simulation data in a fully automated way [26].

The local atomic volume of each atom was calculated by means of the Voronoi tessellation method [27]. We define the free volume of the GB atoms as the difference between the average atomic volume of all GB atoms and the average atomic volume within a fcc single crystal of random solid solution and a chemical composition identical to the composition of the GB.

Samples were quenched from annealing temperature to 300 K. Then they were deformed by imposing a constant engineering strain rate (10^8^ 1/s) in uniaxial direction on the simulation cell. The cell size was allowed to relax perpendicular to the strain axis.

## Results

### Structural characterization

**Figure 1:**
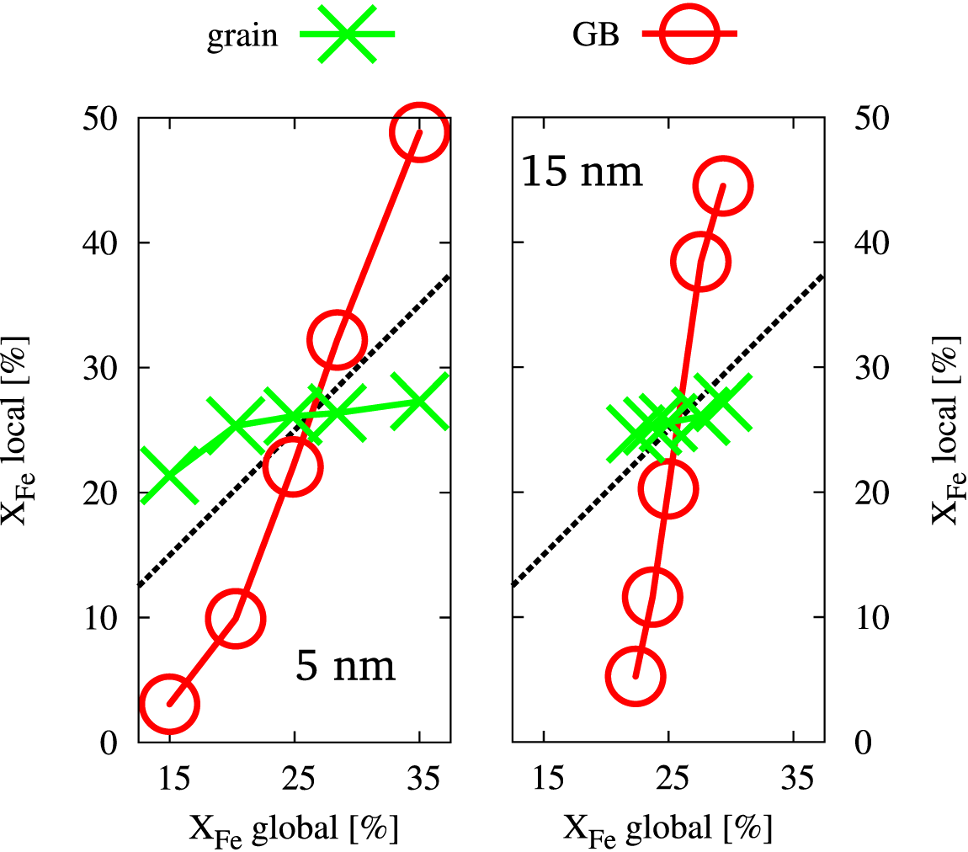
Local composition of the grain interior and the GBs as a function of the global composition for two different grain sizes (5 nm and 15 nm).

**Figure 2:**
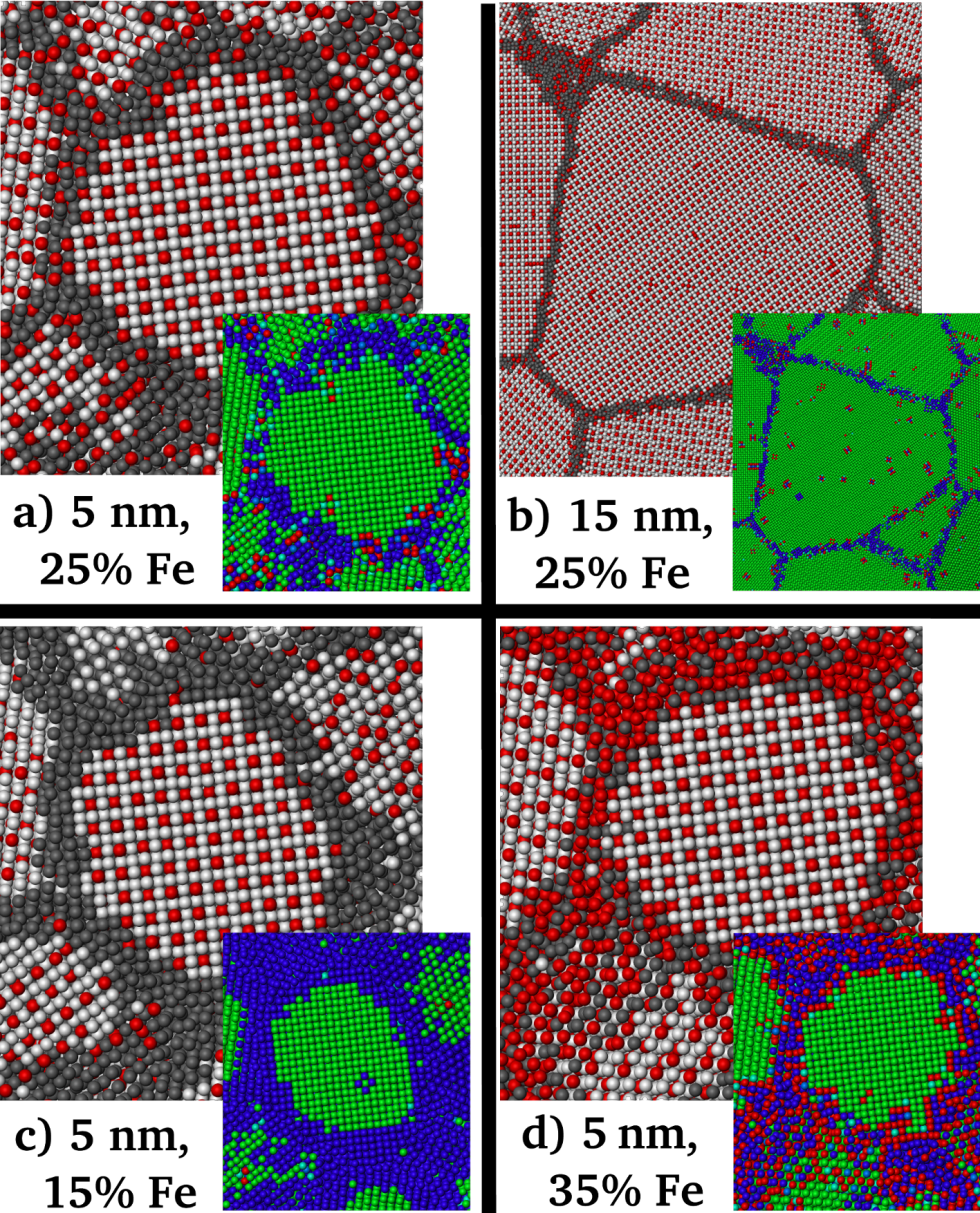
Atomic configurations after annealing with the hybrid MD/MC scheme. Ni atoms in the grain interior are white, Ni atoms in the GB are dark grey, Fe atoms are red. In the inset, the color coding is according to the deviation from perfect L1_2_ short range order, where green refers to perfect L1_2_ structure, while red and blue refer to a positive and negative deviation, respectively. a) 5 nm grain size with a global concentration of 25% Fe. b) 15 nm grain size with a global concentration of 25% Fe. c) 5 nm grain size with an (understoichiometric) global concentration of 15% Fe. d) 5 nm grain size with an (overstoichiometric) global concentration of 35% Fe. Snapshots were generated using OVITO [30].

In a first step, the elemental distribution in the model structures relaxed by the hybrid MD/MC scheme were analyzed in order to quantify the amount of ordering in the grain interior. At finite temperature the instantaneous element distribution is fluctuating and it is therefore useful to introduce the concept of an “effective atom“ [28], namely the average occupancy of an atomic position. By taking the ensemble average over a large number of MC steps, the site occupancy for each atomic position was computed in the semi-grandcanonical ensemble. Figure 1 shows the local site occupancy for the different structural elements (grains and GB), which were identified according to CNA. It can be seen that the chemical composition of the GBs is strongly dependent on the global composition, whereas the grain interior is less affected and stays close to the stoichiometric composition of the L1_2_ structure (25% of Fe). The GB therefore acts as source or sink for solutes accommodating any excess of Fe or Ni, respectively. This allows the grain interior to stay in the ordered and stoichiometric state. Furthermore, it can be seen, that the change in GB composition shows a stronger dependence on the global composition for the case of a 15 nm grain size. Since the GBs need to accommodate the excess in either Ni or Fe, this can be explained by the smaller volume fraction of GB atoms as compared to the 5 nm case. For the atomic arrangement inside the grains we find L1_2_ ordering, irrespective of global composition. It shall be noted, that this finding is independent of the initial configuration prior to equilibration, i.e., whether we start from a perfectly ordered or a disordered state (not shown). Figure 2 shows representative slices through individual grains and the surrounding GBs for different grain sizes and composition. In the inset, the slice is colored according to the deviation from perfect L1_2_ short range order, where green refers to a perfect L1_2_ structure. For all atoms in the grain interior perfect L1_2_ short range order (and consequently also long range order) is conserved for both studied grain sizes. Furthermore, it is visualized (for the 5 nm case), that also for a deviation from stoichiometric composition, the grain interior stays ordered while the GB accommodates the excess in either Ni or Fe. Interestingly, for the maximum deviation from stoichiometric composition, the GB is either almost free of Fe or enriched up to 50%, respectively (Figure 1). It shall be noted that some of the compositions reported here, which show an ordered L1_2_ structure inside the grains, lie outside the phase field of the L1_2_ structure (at 600 K) in the bulk phase diagram reproduced by the interatomic potential [20]. The stability range of the ordered L1_2_ structure in the phase diagram is therefore widened at reduced average grain sizes, where the grains stay ordered and the GBs act as a buffer layer. The experimentally observed disordered state of nc Ni–Fe with an Fe content of up to 28% [5] produced by electrodeposition is therefore considered to result from the route of processing, leading to a kinetically trapped disordered elemental distribution and not due to a differing stable configuration for the case of a nanometer grain size as observed for other systems [29].

### Random alloy: fixed GB composition, varying grain composition (15 nm)

For coarse grained material, the strengthening effect of substitutional solutes (i.e., solid solution strengthening) is well understood, where an increase in strength according to *τ_c_* ~ 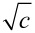 is expected [31]. In order to investigate, whether substitutional solutes in the grain interior have an effect on the macroscopic mechanical properties for the case of a nc solid solution, samples with differing composition inside the grains but identical GB (composition and structure) were compared. The initially equilibrated structure was identical for all cases with a concentration of 20% Fe in the GBs. Different amounts of Fe (5%, 15% and 25%) were then distributed randomly in the grain interior and equilibrated via successive MD steps at 300 K and zero hydrostatic pressure prior to deformation.

**Figure 3:**
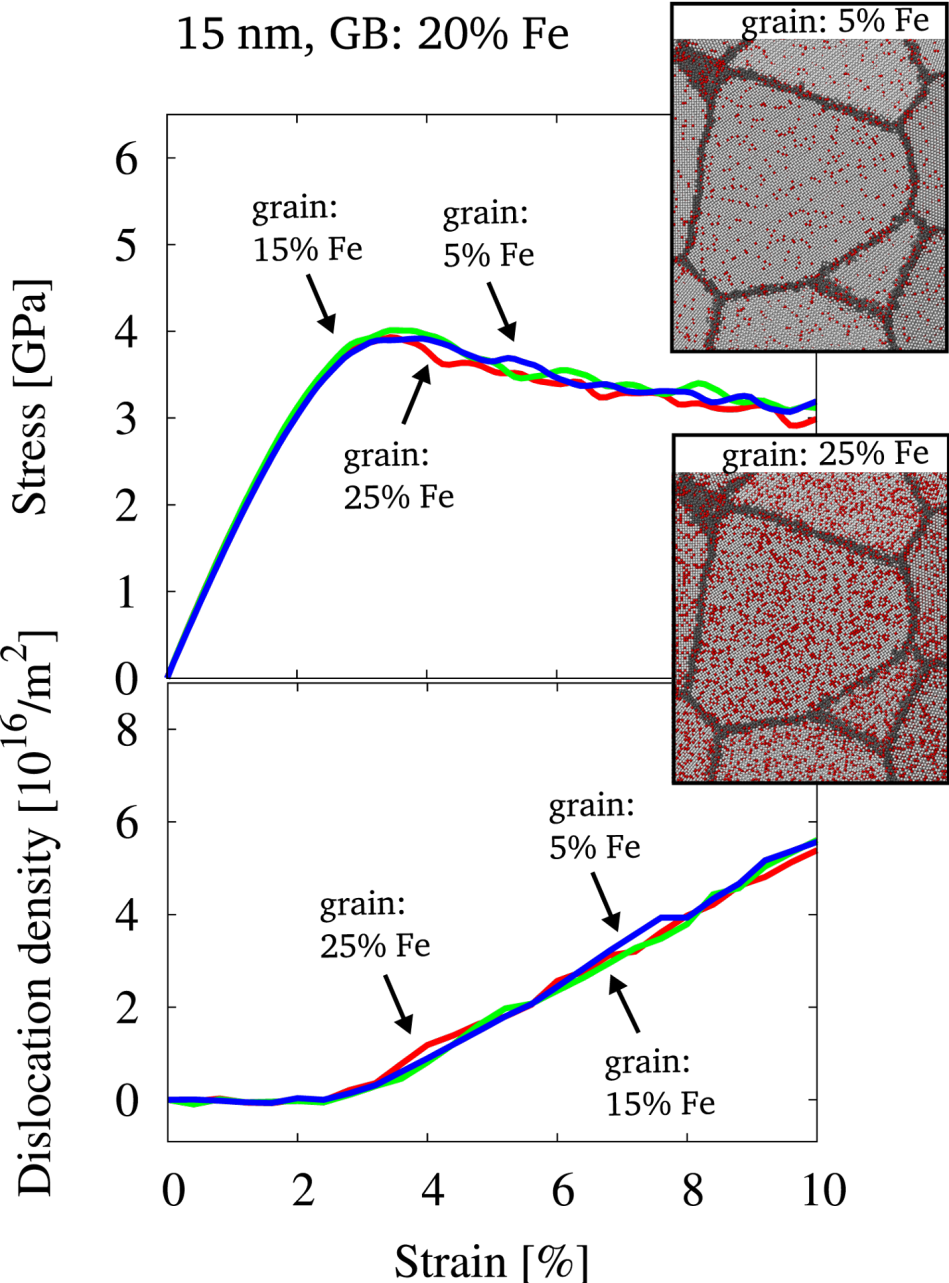
Stress-strain behavior and dislocation density for structures of 15 nm grain size, which were equilibrated by the hybrid MD/MC scheme at a global composition of 25% Fe. The distribution of solutes in the grain interior only was then randomized and the composition inside the grains was changed prior to deformation. The data for three different concentrations inside the grains is shown. Additionally, two snapshots of the initial configuration visualize the solute distribution for two different compositions. Color coding is identical to Figure 2.

Figure 3 shows slices through the initial microstructure for two different compositions, where the random distribution and differing amount of solute in the grain interior is visualized. Figure 3 furthermore shows the stress–strain behavior and the evolution of dislocations in the microstructure under tensile load. It can be seen that a differing composition of the grain interior but identical GB structure (and composition) has little effect on the macroscopic behavior. Neither the stress–strain behavior nor the dislocation density show a dependence on the amount of solute, distributed randomly in the grain interior, even though dislocations are the major carrier of plasticity under the given conditions [32]. We conclude that for the presented case stresses required for dislocation nucleation from the GBs are so high that pinning by solutes inside the grains does not provide an additional barrier for dislocation motion. The stresses required for dislocation nucleation might, however, depend on the state and composition of the GBs. To test a potential effect on the macroscopic properties, we focus on the GB composition in the next section.

### Random alloy: varying GB composition, fixed grain composition (15 nm)

**Figure 4:**
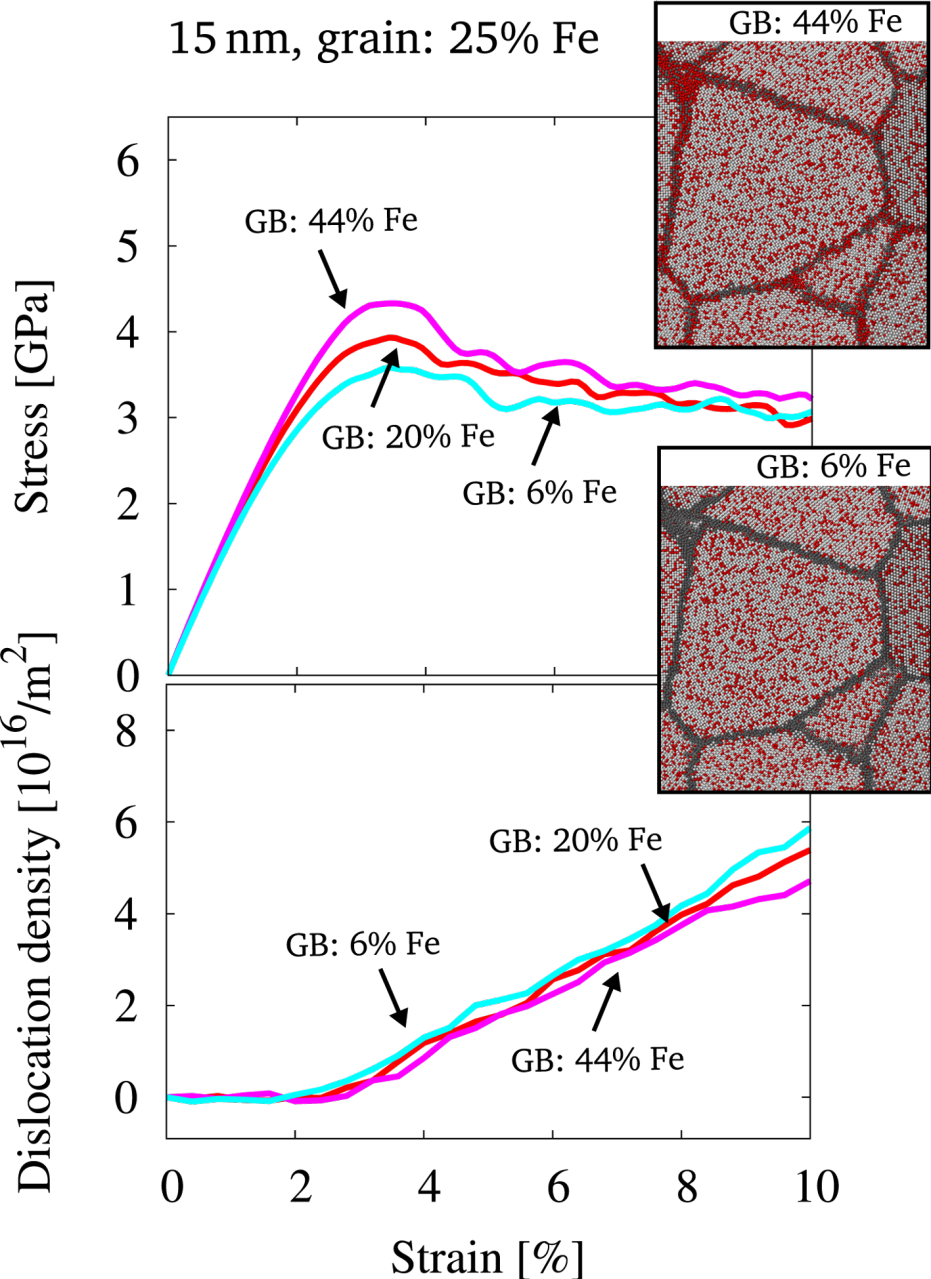
Stress-strain behavior and dislocation density for structures of 15 nm grain size, which were equilibrated by the hybrid MD/MC scheme at different global compositions (22, 25 and 29% Fe). The distribution of solutes in the grain interior only was then randomized with a constant composition of 25% inside the grains prior to deformation. The data for three different concentrations in the GB is shown. Additionally, two snapshots of the initial configuration visualize the solute distribution for two different GB compositions. Color coding is identical to Figure 2.

The delicate interplay between solute distribution and mechanical response of this nc alloy was further studied on samples, where the random solid solution inside the grain interior was held at a constant composition of 25% Fe, but different amounts of solute were introduced into the GB during equilibration. That is, the nc structures were equilibrated via the MD/MC scheme at differing global compositions, leading to the described variation in the GB composition (Figure 1). For all studied samples, the grain interior stays close to the stoichiometric composition after equilibration via the MD/MC scheme (Figure 1). To eliminate any effect due to the slight variation in grain composition and to allow for dislocation processes, the grain interior in all samples was manually brought to a random solid solution with an identical composition for all structures. Prior to deformation, the structures were equilibrated via MD steps at 300 K and zero hydrostatic pressure. Since the composition and relaxation state of the grain interior is identical for all samples, any differences in the macroscopic behavior must result from the changes in GB composition. Figure 4 shows snapshots of a representative grain and the surrounding GBs inside the microstructure for two different cases, where a similar distribution of solutes is present inside the grains, but the GB composition is varied. The corresponding stress–strain behavior (Figure 4) reveals that there is a dependence on the amount of solute added to the GB via the MD/MC scheme, while the evolution of dislocations stays similar for all cases and is therefore not affected. The effect of different amounts of solute in the GB is more pronounced for the onset of plastic deformation. This is consistent with other studies, where it was reported that during the initial stages of deformation, GB plasticity contributes more significantly to the overall plastic deformation [32].

We conclude that the amount of solutes inside the GB or the equilibration state of the GBs is of critical importance for the strength of this nc alloy, which was observed also for completely miscible systems [18]. After demonstrating the important role of the GB equilibration state, we want to discuss the case, where deformation processes are restricted to the GBs. Therefore, we will focus on samples with an ordered grain interior in the next section. Here, dislocation motion in the grain interior is suppressed by the presence of the intermetallic phase.

### Ordered alloy: varying GB composition, fixed grain composition

#### 15 nm grain size

**Figure 5:**
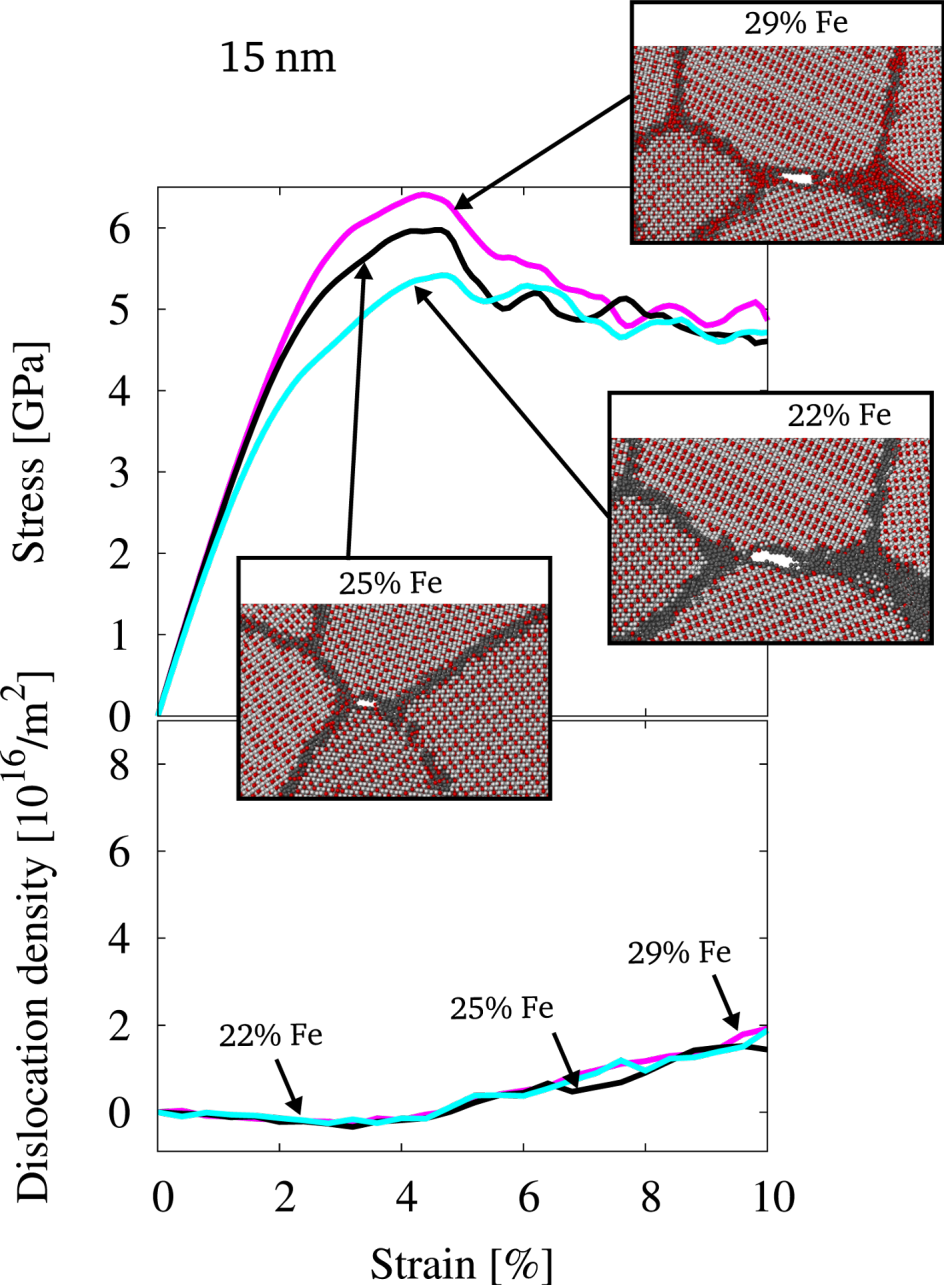
Stress-strain behavior and dislocation density for structures of 15 nm grain size, which were equilibrated by the hybrid MD/MC scheme at different global compositions (22, 25 and 29% Fe). The snapshots of the atomic configuration at the according strain visualize the onset of intergranular fracture. Color coding is identical to Figure 2.

In a next step samples with an ordered grain interior with fixed composition were tested. Here, dislocation processes can be excluded, since the nucleation of superdislocations cannot be expected for the presented strain rates and no fcc-like slip planes are available in the L1_2_ structure. The structures with an ordered grain interior as obtained by equilibration via the MD/MC scheme are therefore expected to deform mainly by GB mediated processes. Figure 5 shows the stress–strain behavior and evolution of dislocations under tensile load for the structures of 15 nm grain size and varying composition. Obviously, the stress–strain behavior shows a clear dependence on GB composition, while dislocation activity is suppressed. The yield stress is also here increased for the case of a structure with GBs enriched in Fe and decreased for a structure with GBs fully depleted in Fe. This is similar to the case of a disordered grain interior as reported above, but even more pronounced.

At the onset of plastic deformation, however, intergranular fracture occurs within the presented structures. This is visualized in the snapshots shown in Figure 5, where slices through the atomic configuration are presented for various stages of deformation. Apparently, suppression of dislocation activity inside the grain interior leads to intergranular fracture, since GB mediated processes cannot be accommodated in samples with relatively large grains (15 nm).

#### 5 nm grain size

**Figure 6:**
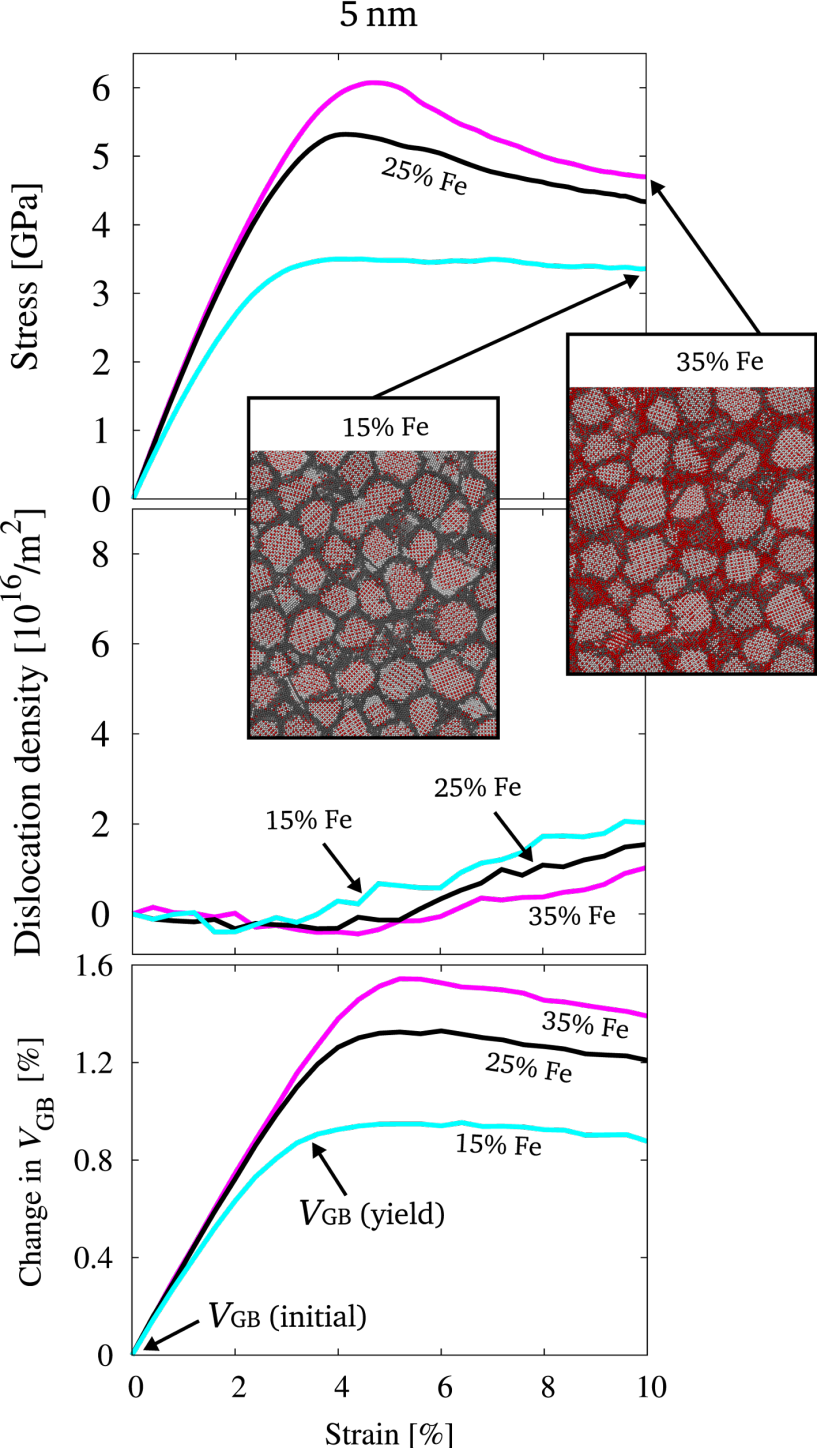
Stress-strain behavior, dislocation density and evolution of GB volume for structures of 5 nm grain size, which were equilibrated by the hybrid MD/MC scheme at different global compositions (15, 25 and 35% Fe). The representative slices through the microstructure at a total strain of 10% visualize, that no intergranular fracture occurred. Color coding is identical to Figure 2.

Since the suppression of dislocation activity leads to intergranular fracture for the 15 nm grain size, the data for the 5 nm grain size is presented here, where a larger contribution to plastic deformation by GB mediated processes can be expected. Figure 6 shows the corresponding stress–strain behavior, evolution of dislocations and change in GB volume under tensile load. Representative slices through the microstructure at 10% total strain are shown, which indicate that intergranular fracture did not occur for the structures with a grain size of 5 nm. From the stress–strain behavior, we find a strong dependence on the GB composition. The dislocation density reveals, that this is not caused by the evolution of intragranular defects, what is consistent with expectations based on the small grain size. It has been shown for other material systems, that the energetic state of the GB, which is closely related to the GB free volume drastically influences the macroscopic mechanical properties of nc microstructures [18–19]. Monitoring the state of the GB for the presented samples in terms of the change in GB free volume (*V*_GB_) shows that there is a strong similarity between the evolution of the GB volume and the stress during straining.

**Figure 7:**
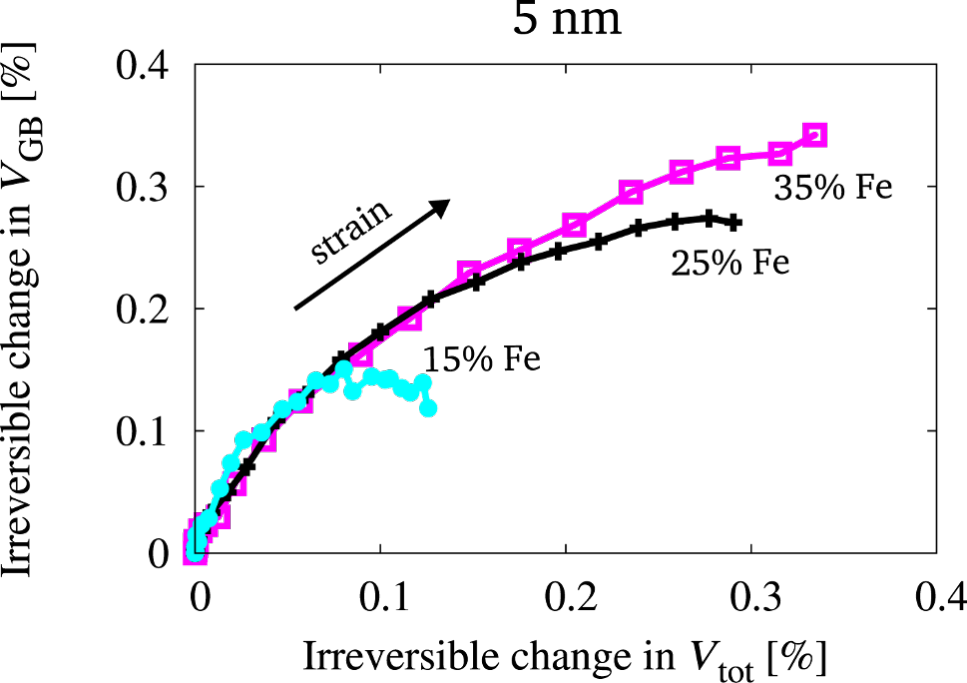
Irreversible change in GB volume (*V*_GB_) as a function of the irreversible change in total sample volume (*V*_tot_) measured at different stages of deformation (every 0.4% of total strain) after unloading. The data for structures of 5 nm grain size, which were equilibrated by the hybrid MD/MC scheme at different global compositions (15, 25 and 35% Fe) is shown.

Obviously, for the structures with a higher yield stress, the GB volume needs to increase more drastically. This increase in GB volume is not only caused by elastic straining but by a general and irreversible increase of the GB volume and therefore also the sample volume. To quantify the amount of irreversible volume change during straining, the samples presented in Figure 6 were unloaded at different stages during deformation (every 0.4% of total strain). The GB volume as well as the total volume was then computed for the unloaded state of the samples. Figure 7 shows the irreversible change in GB volume (*V*_GB_) as a function of the irreversible change in sample volume (*V*_tot_) for three different samples of 5 nm grain size. It can be seen, that the total volume as well as the GB volume of the unloaded structures increase as a consequence of the deformation. It furthermore becomes evident, that the relative change in GB volume is higher than the relative change in the total volume. This means, that the volume of the grain interior is less affected by the overall deformation, which is consistent with GB mediated deformation processes. Comparing the data for the three different structures visualizes, that the irreversible change in GB volume (as well as in total volume) is the highest for the structure with the highest yield stress. Obviously, here, the free volume in the GBs must be raised more significantly in order to deform plastically. This leads to a stronger resistance to plastic deformation and therefore to an increased yield strength.

### Controlling the strength - state of the GB

**Figure 8:**
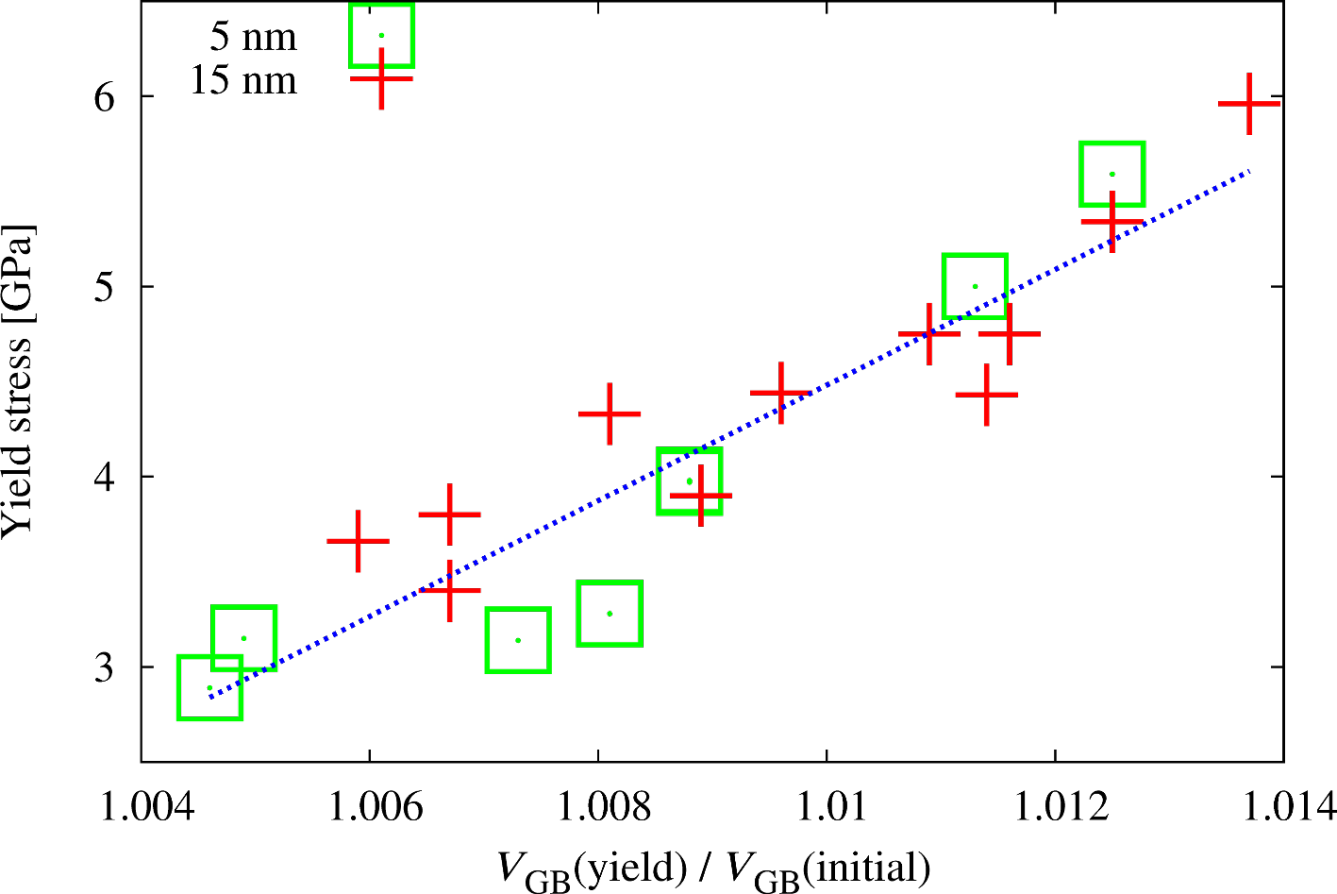
Correlation between yield stress (stress at plastic strain of 0.7%) and the change in free volume in the GBs during deformation for 5 and 15 nm grain size and different states of relaxation. Open squares refer to the data for 5 nm grain size and crosses to the data for 15 nm grain size. (Also data for samples, which were not discussed in detail is shown here.)

For quantifying the relaxation state of the GB, we compute the change in free volume in the GBs during straining for all structures. The correlation between the yield stress of the material and the change in GB free volume at the yield point is analyzed in order to quantify the relation between the onset of plastic deformation and the increase in free volume in the GBs. Experimentally, it was observed, that the 0.2% offset cannot be used as a yield criterion for nc metals (nc Ni–Fe) [12]. From in-situ X-ray peak profile analysis it was reported, that the transition from micro- to macroplasticity rather occurs around 0.7% for nc Ni [33]. We therefore took the 0.7% offset as a yield criterion for the presented data and extracted the yield stress as the stress at a plastic strain of 0.7%. For the same plastic strain, the change in GB free volume was extracted from the data. (It shall be noted, that the results are rather insensitive to the choice of the offset or employing the maxima in the stress–strain behavior as a criterion for yielding.) Figure 8 shows the correlation between the yield stress and the change in free volume in the GB. Obviously, the data for different stages of equilibration and different stages of ordering falls on the same trend. Remarkably, even the data for different grain sizes lies on the same trend, irrespective of the state of the grain interior. (The data for structures, which were not discussed in detail is also shown.) It becomes evident, that the state of the GB and therefore the necessary increase in GB free volume controls the yield strength of the material. This is consistent with observations made for miscible systems [18] and observations on materials where dislocation slip is completely inactive (ultrananocrystalline diamond), where it was observed, that the yield stress scales with the stress required for GB sliding [34].

## Conclusion

For studying plasticity in nc alloys with an ordering tendency, we performed hybrid MD/MC simulations of nc Ni–Fe of different grain sizes and compositions. We have shown that a nanocrystalline microstructure widens the stability range of the ordered L1_2_ phase. Furthermore we could proof that conventional solid solution hardening is absent. For the presented conditions and samples, the equilibration state of the GBs controls the strength of the material, irrespective of the grain size.

The employed algorithm allowed us to obtain the equilibrium solute distribution for a given microstructure. For grain sizes in the nanometer range, ordering in Ni–Fe alloys was not observed experimentally [5] and it is reported, that nc materials can have a different ground state crystal structure than coarse crystalline material at the same composition and temperature [29,35]. For the case of Ni–Fe, however, our simulations reveal that the ground state of this alloy (at an Fe content of 25%) is the L1_2_ ordered phase also for a 5 nm grain size. Equilibrating a nc model structure by the hybrid MD/MC scheme leads to an ordered grain interior, irrespective of the starting configuration. We can thus conclude that the experimental preparation procedure is producing metastable nc structures in the chemically disordered state. Our simulations furthermore show that decreasing the grain size into the nanometer range is extending the compositional phase field of the L1_2_ structure, where perfect ordering in the grain interior is observed, since the GBs act as buffer layer accommodating excess components. The segregation of either Ni or Fe to the GB is therefore energetically in favor as compared to the loss of order inside the grains.

For studying the influence of solute distribution on the mechanical properties, additional configurations of solute distribution were obtained by randomly distributing the solute within individual parts of the microstructure, e.g., the GBs or grain interiors. Successive uniaxial straining simulations for the different samples then allowed to evaluate the influence of individual effects. For the structures with an equilibrated GB and a random solid solution of varying composition inside the grain interior we find that there is little effect of the composition of the grain interior on the macroscopic mechanical properties. The dislocation extraction algorithm [26] reveals that there is a considerable amount of dislocation activity inside the grains. It is, however, concluded that solid solution hardening is not present in the described structures since the stresses necessary for GB sliding and dislocation nucleation obviously exceed the stresses required for dislocation motion in the presence of substitutional solutes. For the structures with an equilibrated GB of varying composition and a random solid solution with constant composition inside the grains, we observe that the composition of the GB has an effect on the macroscopic mechanical properties. Here, an excess in Fe strengthens the structure, while a depletion in Fe decreases the strength. This is consistent with findings for miscible systems, where a maximum in the strengthening effect of solutes was observed for intermediate compositions [18]. Also in this case, the dislocation density inside the grains does not change. The differing strength must therefore result from the state of the GB. The drastic increase in dislocation density, however, shows that a large fraction of plastic deformation is carried by intragranular defects. This could explain, why the difference in the response vanishes for larger strains, where intragranular defects contribute more significantly, which is reported also from experiment [12]. Suppressing dislocation motion by leaving the grain interior in the energetically favored, ordered state resulted in an even stronger dependence on GB composition. For the 15 nm grain size, however, an ordered grain interior also resulted in intergranular fracture. The processes in the GB can apparently not accommodate themselves for this grain size. Ductility could here be conserved by increasing the volume fraction of the GBs, i.e., by a smaller grain size. For the 5 nm samples, we find no intergranular fracture during straining and a strong dependence of strength on GB composition. For the 5 nm grain size, where GB mediated processes contribute stronger to plastic deformation, the material stays ductile also for the case of ordered grains. Here, the macroscopic mechanical properties are controlled by the state of the GB only.
